# Evaluating correlates of healthy eating and dietary quality among older adults: a mixed methods approach to development and application of a new survey instrument

**DOI:** 10.3389/fpubh.2025.1661573

**Published:** 2025-10-20

**Authors:** Sara K. Rosenkranz, Donya Shahamati, Anna Biggins, Alissa Mick, Christopher Acosta, Richard R. Rosenkranz

**Affiliations:** ^1^Department of Kinesiology and Nutrition Sciences, School of Integrated Health Sciences, University of Nevada, Las Vegas, NV, United States; ^2^Department of Food, Nutrition, Dietetics and Health, College of Health and Human Sciences, Kansas State University, Manhattan, KS, United States

**Keywords:** behavioral assessment, instrument development, nutrition barriers, psychometric evaluation, healthy aging, eating motivation

## Abstract

**Objectives:**

Community-dwelling older adults face unique challenges related to nutrition and health, but little is known about their barriers and facilitators for healthy eating behaviors. This study sought to develop and evaluate a new instrument to measure the capability, opportunity, and motivation for healthy eating behaviors (COM-HE) among community-dwelling older adults.

**Design:**

A mixed methods approach was used to obtain qualitative and quantitative data. Participants were aged 65 years or older, community-dwelling, and English-speaking. Participants engaged in focus groups (*n* = 12) and pilot-testing (*n* = 81) to evaluate the COM-HE instrument. The Rapid Eating Assessment for Participants – Shortened Version (REAP-S) questionnaire was utilized to examine correlations between the COM-HE instrument and self-reported dietary quality. Descriptive and inferential statistics were used to investigate acceptability, reliability, and validity.

**Results:**

The COM-HE instrument achieved acceptable internal consistency (Cronbach’s alpha = 0.847–0.986), displayed varying levels of uni-dimensionality based on multiple principal component analyses (total variance explained by three components = 86.72%), and was correlated with self-reported dietary quality scores (*r* = 0.409, adjusted *R*^2^ = 0.099, *p* = 0.031). Preliminary data suggest that the scale was acceptable in terms of readability and understanding among a convenience sample of generally well-educated older adults.

**Conclusion:**

The new COM-HE instrument was acceptable, reliable, and valid among a homogeneous sample of adults over 65 years of age. These results suggest a need for additional development, evaluation, and refinement of the instrument in more diverse groups of older adults.

## 1 Introduction

**Figure 1:**
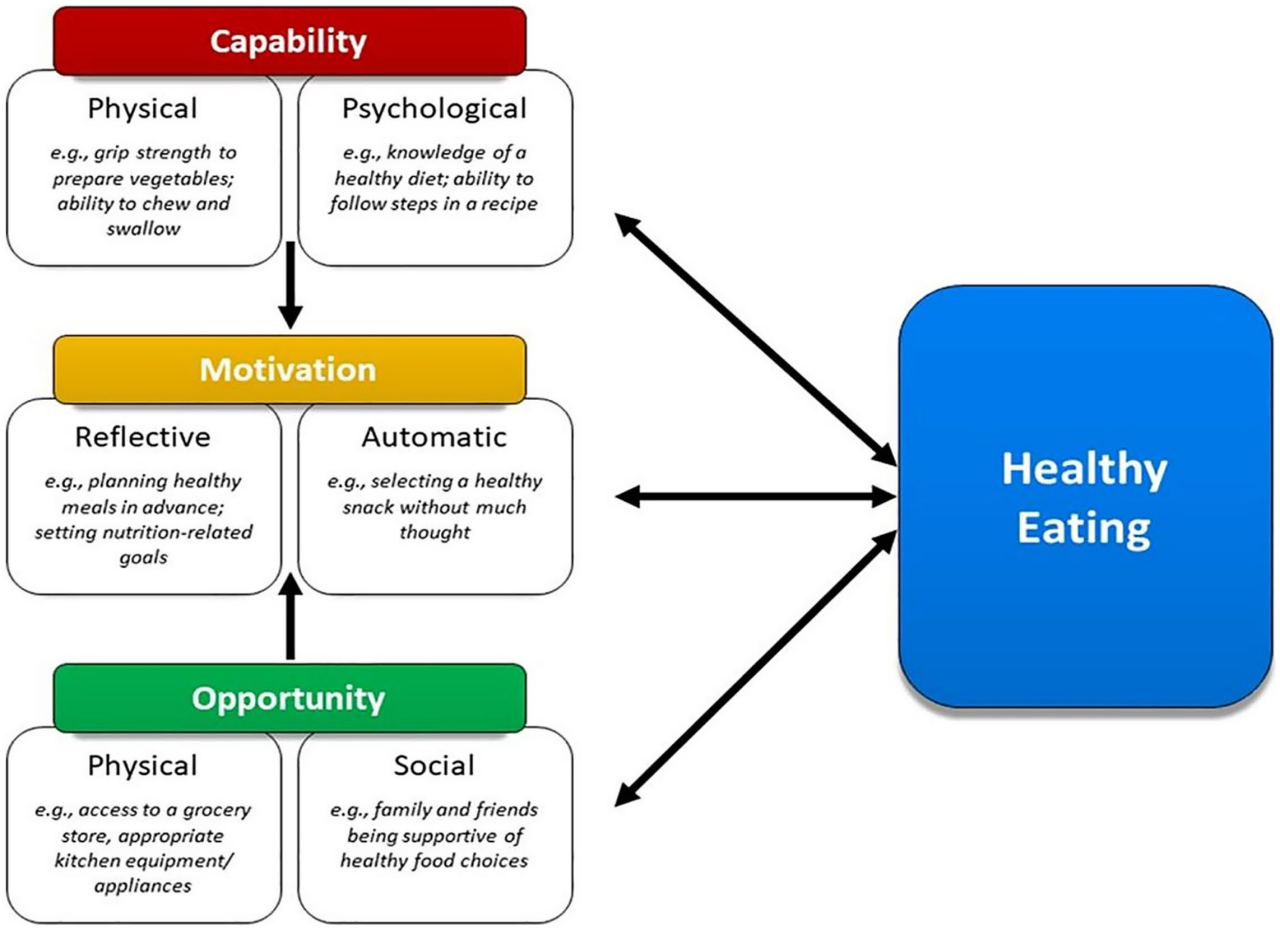
Framework illustrating how capability, opportunity, and motivation influence healthy eating behaviors in older adults.

**Figure 2:**
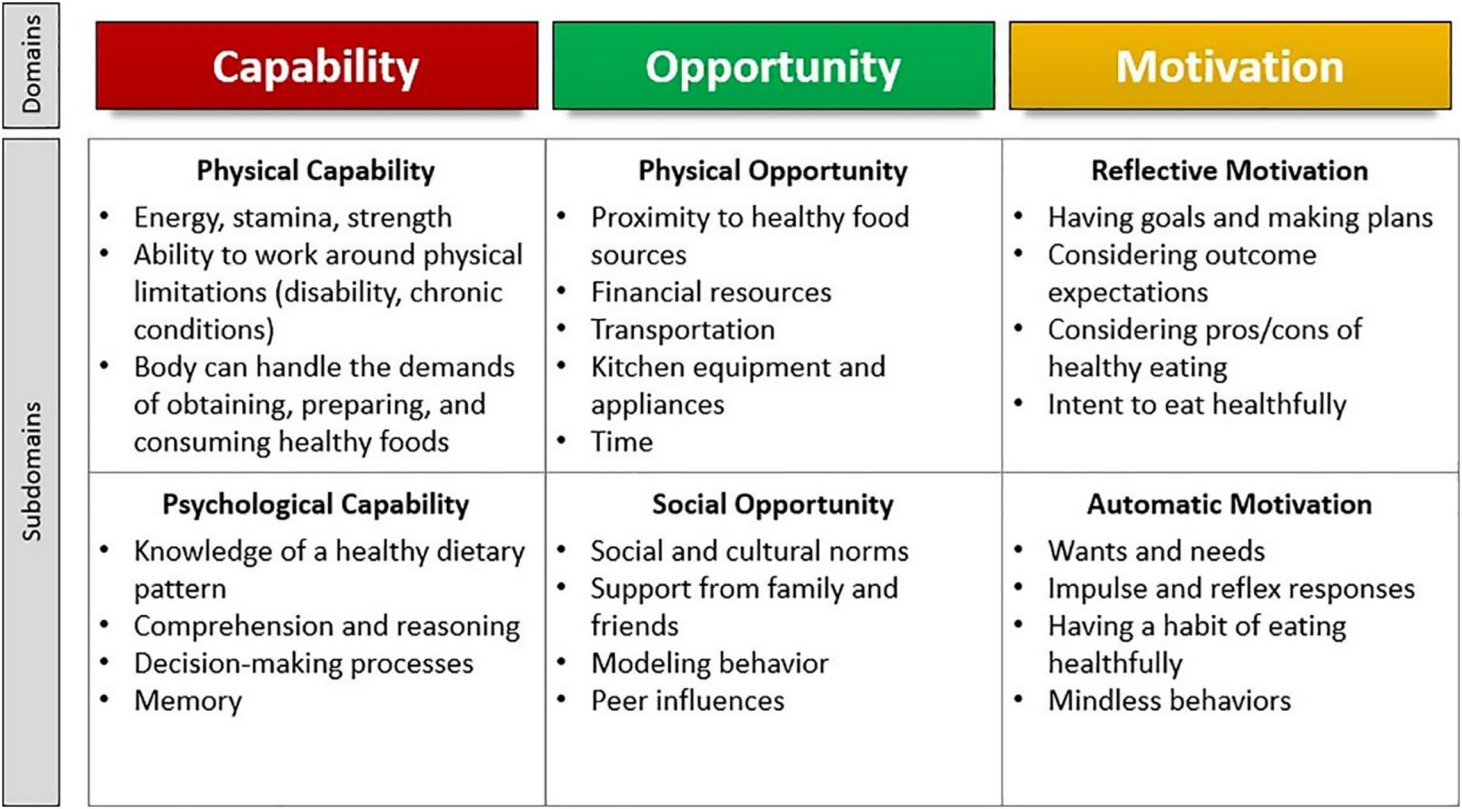
Illustration of the COM-HE (Capability, Opportunity, Motivation for Healthy Eating) framework outlining key domains and subdomains influencing healthy eating behavior among older adults.

Approximately 80% of adults over the age of 65 are living with at least one chronic condition, and one-third of older adults experience limitations in activities of daily living, which includes preparing meals (1). Although age is a non-modifiable risk factor for chronic diseases such as diabetes, cardiovascular diseases, arthritis, respiratory diseases, and cancers, nutrition represents a modifiable factor for maintaining and improving functionality and quality of life among the older adult population (2, 3). Furthermore, higher dietary quality is associated with lower risk of developing limitations in activities of daily living and depression, and more favorable health outcomes such as reduced risk of hypertension, improved glycemic control and cognitive function, and better self-rated health and quality of life (4–7). Nutrition behaviors are not determined solely by intrapersonal factors such as self-efficacy, beliefs, and attitudes toward nutrition, but are also influenced by social and environmental factors outside of the individual. When choosing appropriate behavior change interventions, those that consider multiple factors may be more effective (8). One framework that appropriately describes these interwoven aspects of nutrition behaviors is the Behavior Change Wheel (BCW). The BCW includes three core domains that interact to produce behavior: capability, opportunity, and motivation, referred to as the COM-B model. Each of the three core domains comprises two subdomains: Capability (physical and psychological); Opportunity (physical and social); and Motivation (reflective and automatic) (Figure 1). While several other behavioral theories and frameworks have been used to study health behaviors, largely, there has been a focus on individual-level factors that may not fully capture the broader influences on eating behaviors, particularly in older adults. The COM-B model offers a comprehensive and flexible framework that integrates physical and social opportunities, psychological and physical capabilities, and both reflective and automatic motivation (Figure 2). These components are especially relevant to older adults, particularly those who are community-dwelling, whose environments and capabilities may be quite variable and whose dietary behaviors may be shaped by factors such as social isolation, physical limitations, habits and longstanding beliefs, and emotional eating.

The six COM-B subdomains can be applied to identify barriers, facilitators, and other modifiable factors for eating behaviors toward improving health (9). While a general, six-item COM-B questionnaire was developed by Keyworth and colleagues, to our knowledge, no instruments exist for evaluating capability, opportunity, and motivation for nutrition-related behaviors within the older adult population (10). Measurement of facilitators and barriers for healthy eating among community-dwelling older adults using the COM-B model components, represents an important gap in the available research literature. Despite the growing emphasis on promoting healthy eating behaviors among older adults, there is a need for a valid instrument that can be adapted to various contexts and used to assess the behavioral correlates of healthy eating in this population. Such an instrument will provide a structured framework to capture the interplay between capability, opportunity, and motivation for influencing healthy eating behaviors. With these considerations in mind, the primary aim of the current study was to develop a new instrument to measure the correlates of healthy eating behaviors in older adults, guided by the COM-B model. Secondary outcomes of the instrument development process included evaluating psychometric properties including validity, reliability, and acceptability of the new instrument; and examining associations between the COM-B model components and self-reported dietary quality among community-dwelling older adults. Developing such an instrument for use among researchers and health professionals could assist with identifying appropriate behavioral nutrition interventions for this population, thus leading to improved dietary quality and health outcomes for community-dwelling older adults.

## 2 Methods

### 2.1 Study design

**Figure 3:**
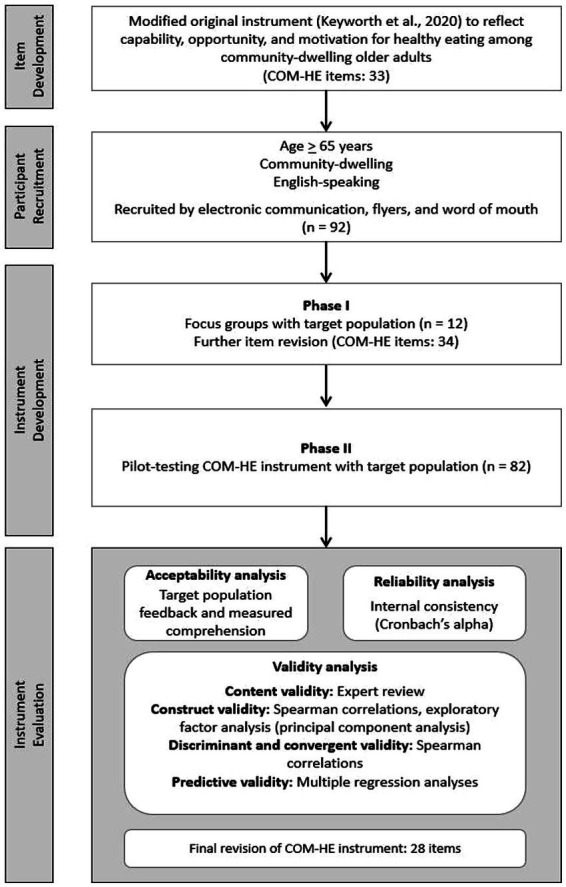
Overview of the development and testing process for the COM-HE (Capability, Opportunity, Motivation for Healthy Eating) instrument, including item refinement, pilot testing, and analyses for acceptability, reliability, and validity.

A mixed methods approach was used to collect both qualitative and quantitative data. Study design was informed by the three phases for best practices in scale development, which included item development, instrument development, and instrument evaluation (11). Item development differed from instrument development in that item development involved generation of individual questions that had potential to be included in the subdomains within the new instrument; instrument development involved compiling those items into one concise, cohesive instrument using an online survey platform. The study methods are presented according to the chronological order in which they occurred over the timeline of the study, beginning with item development and progressing to participant recruitment, instrument development, and instrument evaluation. The process of instrument development activities is described in Figure 3.

### 2.2 Item development

#### 2.2.1 Original items

The Keyworth questionnaire was modified to further evaluate the capability, opportunity, and motivation for healthy eating behaviors (COM-HE) among community-dwelling older adults (10). The COM-HE instrument included the same six subdomains as the original instrument. The purposes of each of the six subdomains were discussed by a panel of three subject matter experts (SKR, RRR, AB) and definitions were generated to clarify the meaning of each subscale for this study. Collaboration and practical expertise in nutrition, gerontology, and behavior change were imperative for the thoughtful expansion of each of the six subdomains to reflect the facilitators and barriers for nutrition-related behaviors unique to community-dwelling older adults. We operationally defined the term healthy eating for the purpose of the current study. The healthy eating definition was determined by referencing national and international recommendations for the components of dietary patterns that are conducive to positive health, including those from the 2015–2020 Dietary Guidelines for Americans (12), the World Health Organization (13), and previous literature on the social and cultural aspects of eating behaviors (14). The full definition for “healthy eating” can be found in the introduction section of the COM-HE instrument in the Supplementary Appendix. Once the healthy eating subdomains were clearly defined, we developed health-promotion–oriented items to effectively capture the key aspects of each subscale while minimizing participant burden by keeping the survey concise. This initial development process resulted in the generation of five to seven items for each subdomain in the draft COM-HE instrument. The draft instrument underwent continual revision until it was determined to be satisfactory to enter Phase I, which involved gathering feedback from the target population during focus groups. The resulting draft instrument comprised 33 items and was entered into Qualtrics (Qualtrics, Provo, UT), a widely user-friendly online survey software platform (Supplementary Appendix).

### 2.3 Participant recruitment

**Figure 4:**
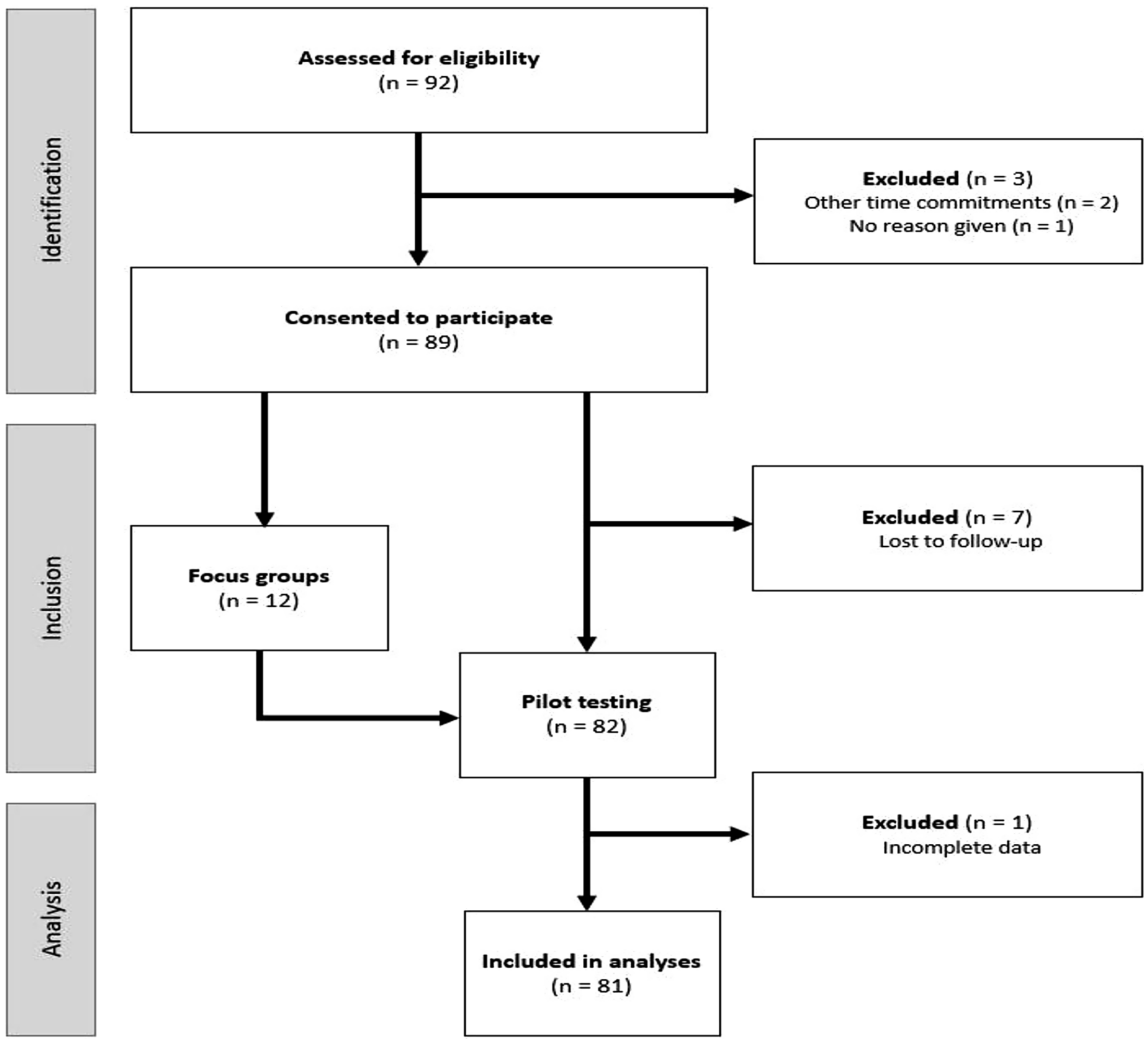
Flow of participants through the study, from eligibility assessment (*n* = 92) to final analysis (*n* = 81), with exclusions noted at each stage. n = number of participants.

Participant recruitment was conducted in two phases, referred to as Phase I: Focus Groups, and Phase II: Pilot Testing. The current study was approved by the Kansas State University Institutional Review Board and received approval number 10911. A convenience sample of eligible participants was obtained through electronic communication, flyers in public spaces, and word of mouth in the Riley County, Kansas area. Eligible participants were: (1) aged 65 years or older, (2) community-dwelling (e.g., not residing in a nursing home, assisted living community, memory care facility, etc.), and (3) English-speaking. The inclusion criteria were intentionally kept as broad as possible for the purpose of gaining a representative sample of the local population. Individuals who were interested in the study were invited to initiate email correspondence with the researcher to receive additional information and confirm eligibility requirements. Verbal and written informed consent were obtained prior to study participation. All participants were entered into a raffle for one of 40 $25 grocery store gift cards upon completion of the study. Twelve participants participated in focus groups to evaluate the draft COM-HE instrument during Phase I. All focus group participants also chose to participate in Phase II, during which they completed the revised instrument. An additional 80 participants expressed interest in Phase II, bringing the total number of Phase II participants to *n* = 92. Out of these individuals, 82 completed pilot-testing, 81 provided complete data and were included in data analysis, and 44 completed the instrument within 14 days of the first assessment. A diagram of recruitment flow can be found in Figure 4.

### 2.4 Instrument development

#### 2.4.1 Phase I: focus groups

The purpose of conducting focus groups with members of the target population was to ensure participant comprehension of items, uncover any problematic items, and determine whether the items elicited the intended information (15). Involvement of the target population in item development can help establish face validity, or whether an instrument appears to measure what it aims to measure on the surface (16). Based on general focus group recommendations of having no less than four and no more than 12 participants per focus group (17), we aimed to include five to eight participants per focus group with a total of 20–30 participants in Phase I. With that aim in mind, four focus group sessions were held in person at the Physical Activity and Nutrition Clinical Research Consortium (PAN-CRC) lab, virtually via Zoom, and through a hybrid of in-person and virtual groups. During the focus group sessions, the draft COM-HE instrument was displayed on a screen and participant feedback was elicited through the use of semi-structured interview questions asked by one researcher (AB). Drawing on recommendations, the predetermined list of interview questions reflected a modified form of cognitive interviewing, using techniques that the researcher had been trained in (18). This approach involved the use of retrospective and paraphrasing techniques with the flexibility to ask probing questions to elicit additional clarifying information. Participant comments were typed into a spreadsheet by three trained research assistants. No audio or video recordings were collected.

Suggestions from participants were considered and some were tested throughout this focus group phase to gauge acceptability of items that were revised or added. For example, participants in the first focus group suggested making a change to the item “I want to practice healthy eating to improve my overall health,” to reflect the fact that some older individuals might have a goal of maintaining, rather than improving, their overall health. Consequently, a new item was created to reflect this suggestion, and participants in the remaining focus group sessions were shown both the original and the modified version of the item to provide feedback on the proposed change. Participants were also asked to provide their general format-related preferences for completing online questionnaires, such as only one question being displayed at a time, being able to use “back” buttons to access previously answered questions, and the presence of a progress bar.

#### 2.4.2 Phase II: pilot-testing with target population

The pilot-testing phase involved administering the revised COM-HE instrument (34 items) to participants at two time points, approximately 2 weeks apart to determine test–retest reliability of the COM-HE instrument, and administering the REAP-S questionnaire at the first time point to obtain a measure of self-reported dietary quality. Both instruments were administered via Qualtrics, and participants received a link via email to complete the surveys. Reminders were provided approximately 3 days following the initial email if the survey responses had not yet been recorded. The REAP-S questionnaire formatting was modified slightly for easier application within Qualtrics, and two items were changed to more appropriately reflect current nutrition guidelines. Of the two modified items, the first evaluated dairy consumption, and the phrase “or dairy alternatives” was added to encompass current trends in the presence of dairy alternatives in the U.S. food supply (19). The second modified item evaluated snack food consumption, which included a measurement of “regular potato chips, nacho chips, corn chips, crackers, regular popcorn, [and] nuts”––the food item “nuts” was removed from this category of other salty snack foods, as nuts are generally considered to be health promoting (20).

### 2.5 Data analysis

#### 2.5.1 Qualitative analysis

Content analysis of qualitative data was conducted to categorize comments from participants regarding the COM-HE instrument during Phase I and Phase II. Phase I content analysis involved a thorough examination of the verbal comments transcribed for each item of the draft COM-HE instrument after all focus group sessions were completed. The purpose of content analysis was to develop a deeper understanding of participants’ thought processes while reading each item to ascertain comprehension of the concepts the items intended to measure (21). While it is more common in qualitative content analysis to identify salient themes as they emerge during thematic analysis of the text, the process of setting “templates,” or *a priori* codes, is also an acceptable method of qualitative analysis and was useful for the purpose of this study in order to guide discussion of thought processes which can be difficult to describe (22). Three categories were determined *a priori* to help differentiate between the items that appeared to (1) achieve sufficient understanding, (2) achieve moderate understanding, and (3) fail to achieve understanding among the target population. Content analysis in Phase II involved a similar examination of typed comments from the six open-ended questions, one from each subdomain, of the COM-HE instrument. These questions were intended to uncover additional information regarding participants’ apparent understanding of the items within each subdomain. Similar to the Keyworth et al. study (10), participant comments on the items in each subdomain were coded into *a priori* categories of “positive,” “negative,” or “neutral.” Comments were coded as positive if agreement or enjoyment of the items was indicated; negative if confusion or dislike of the questions was expressed; and neutral if the thinking process for answering the questions was explained, personal health or nutrition behaviors were mentioned, or other features of the survey were noted. A summary of Phase II content analysis combined with the quantitative measures of understanding and ease of reading can be found in Table 1.

**Table 1:** Phase II acceptability analysis.

	Participant rating^a^		Comment type			
Acceptability by subscale	Mean	(SD)	Positive	Negative	Neutral	No comment
Physical capability			1 (1.2%)	6 (7.3%)	7 (8.5%)	68 (82.9%)
Ease of reading	9.28	1.10				
Understanding	9.61	0.65				
Psychological capability			1 (1.2%)	8 (9.8%)	5 (6.1%)	68 (82.9%)
Ease of reading	9.30	1.05				
Understanding	9.41	0.91				
Reflective motivation			0 (0%)	2 (2.4%)	13 (15.9%)	67 (81.7%)
Ease of reading	9.43	1.00				
Understanding	9.55	0.88				
Automatic motivation			1 (1.2%)	1 (1.2%)	10 (12.2%)	70 (85.4%)
Ease of reading	9.40	1.04				
Understanding	9.49	0.91				
Physical opportunity			0 (0%)	2 (2.4%)	6 (7.3%)	74 (90.2)
Ease of reading	9.54	0.84				
Understanding	9.63	0.79				
Social opportunity			0 (0%)	2 (2.4%)	10 (12.2%)	70 (85.4%)
Ease of reading	9.50	0.76				
Understanding	9.57	0.73				

a Possible scores ranging from 0 to 10, with 10 indicating greater ease of reading and understanding.

#### 2.5.2 Quantitative analysis

Quantitative data analyses included descriptive and inferential statistics using IBM SPSS Statistics software Version 28. Psychometric testing included the properties of acceptability, reliability, and validity. Chi-square tests of independence (*χ*^2^) were used to evaluate potential differences in demographic variables (age, gender, education, and employment).

##### 2.5.2.1 Acceptability

Acceptability was assessed by determining the readability and understanding of the subscales using two quantitative questions at the end of each subscale: (1) “Overall, did you find the previous set of questions difficult or easy to read?” rated on a Likert type scale from 0 (difficult to read) to 10 (easy to read), and (2) “Overall, how confident are you that you understood what the previous set of questions were asking?” rated on a Likert type scale from 0 (not at all confident) to 10 (very confident). Mean and standard deviation of the ratings were determined and are presented alongside the qualitative analysis in Table 1. An evaluation of floor and ceiling effects was also utilized to gauge acceptability of the scale items within this sample of older adults. Floor and ceiling effects were considered to be present when 15% of participants achieved the lowest or highest possible score on an item or scale, making it difficult to distinguish low-scoring or high-scoring individuals from one another (23). Floor and ceiling effects are generally considered to be undesirable, because these effects can occur when scales lack extreme items on either end of measurement, but can also be present when the respondents are very similar to one another (24, 25).

##### 2.5.2.2 Reliability

Internal consistency, which is a measure of reliability, was assessed using Cronbach’s alpha levels. Alphas were determined for each subscale separately, and for the six COM-HE subscales together. Test–retest reliability was assessed using Intraclass Correlation Coefficients (ICC).

##### 2.5.2.3 Validity

Construct validity was evaluated with the principal component analysis (PCA) method of exploratory factor analysis (26). Uni-dimensionality is ascertained through PCA by testing whether a set of items in a scale measure a single concept or construct (23, 26, 27). Multiple PCA tests were conducted to assess the dimensionality of the COM-HE instrument. The first set of PCA tests examined each subscale separately by loading all of a subscale’s items and extracting one factor per test. This provided an evaluation of the uni-dimensionality, or whether the items in each subscale measured only one component. The six subscales were then loaded together to extract three factors to assess whether the subscales reflected three separate components. Subsequently, paired subscales (i.e., physical + psychological capability; reflective + automatic motivation; and physical + social opportunity) were tested by extracting two factors per test to examine whether each of the three core domains were measuring two separate components. Finally, all 28 COM-HE items (following removal of reverse-scored items) were tested together to extract six factors to examine whether the six separate subscales measured six separate components. Eigenvalues were evaluated to assess the variance that could be explained by the extracted factor(s), using values between 0.70 and 0.95 as acceptable subscale internal reliability (28). Discriminant and convergent validity were assessed using Spearman correlations, as a non-normal distribution was observed with all COM-HE subscales favoring negative skewness (29).

Finally, concurrent validity was assessed using a series of multiple regression models to examine associations between the COM-HE variables and REAP-S scores. The COM-HE subscales were assessed individually for correlations with REAP-S scores, and then paired to assess the C, O, and M scales and REAP-S scores REAP-S scores were determined by adding together the scores for the first 13 items for each participant. Scores could range from 13 to 39, with higher scores reflecting higher dietary quality.

## 3 Results

### 3.1 Participant characteristics

A total of 81 study participants were included in analyses. The mean age of participants was 73.5 years (SD = 6.31), and the majority were female (63%). There was a marked lack of ethnic diversity in this sample, with 100% of participants describing themselves as non-Hispanic White (*n* = 81). The sample was also generally highly educated, with 82.7% of participants holding a bachelor’s degree or higher. Males and females did not significantly differ on education (*χ*^2^ = 5.25, *p* = 0.263) employment status (χ^2^ = 6.24 *p* = 0.101), or age (χ^2^ = 9.02, *p* = 0.061). Participant characteristics are presented in Table 2.

**Table 2:** Participant characteristics.

	Total (*n* = 81)		Male (*n* = 30)		Female (*n* = 51)	
Variable	*N*	%	*N*	%	*n*	%
Gender						
Male	30	(37.0)				
Female	51	(63.0)				
Prefer to self-describe	0	(0)				
Total	81					
Ethnicity						
White	81	(100)				
Other	0	(0)				
Total	81					
Education						
Less than 9th grade	0	(0)	0	(0)	0	(0)
9–12th grade, no diploma	0	(0)	0	(0)	0	(0)
High school or GED equivalent	1	(1.2)	0	(0)	1	(2.0)
Associate’s degree or vocational training	6	(7.4)	1	(3.3)	5	(9.8)
Some college (no degree)	7	(8.6)	1	(3.3)	6	(11.8)
Bachelor’s degree	27	(33.3)	9	(30.0)	18	(35.3)
Graduate or professional degree	40	(49.4)	19	(63.3)	21	(41.2)
Total	81	30		51		
Employment status						
Not working (retired)	62	(76.5)	23	(76.7)	39	(76.5)
Working (paid employee)	12	(14.8)	2	(6.7)	10	(19.6)
Working part time (paid or unpaid)	5	(6.2)	4	(13.3)	1	(2.0)
Working (self-employed)	2	(2.5)	1	(3.3)	1	(2.0)
Total	81	30		51		

	(Total) Mean	(SD)	(Male) Mean	(SD)	(Female) Mean	(SD)
Age (years)	73.54	(6.31)	75.83	(6.65)	72.20	(5.74)

### 3.2 Phase I: focus groups

Focus group sizes ranged from 1 to 5 participants. Content analysis of focus group data indicated adequate acceptability of the draft COM-HE instrument but revealed 13 items that were perceived as problematic among the target population based on the coding criteria outlined for the qualitative analysis. Out of the 33 COM-HE original subscale items and eight item descriptions (definitions provided prior to each of the scale subcomponents) assessed by participants during the focus group sessions, 26 subscale items (79%) and 2 descriptions (25%) achieved sufficient understanding and did not need to be modified, suggesting appropriate face validity of the generated items. Four subscale items (12%) and 3 descriptions (38%) achieved moderate understanding and were then modified slightly. Finally, 3 subscale items (9%) and 3 descriptions (38%) failed to achieve understanding and underwent significant modification before inclusion in the final instrument. A summary of item modification following Phase I is presented in Table 3.

**Table 3:** Content analysis of focus group data.

Category	COM-HE item or description	Resulting modifications	Quotes to support item modification
Items failing to achieve understanding (*n* = 6)	*Healthy eating description:* A diet that reflects healthy eating includes vegetables, fruits, whole grains, low-fat dairy or dairy alternative, seafood, legumes, nuts, moderate consumption of alcohol (up to 2 drinks per day for men; up to 1 drink per day for women) if alcohol is consumed at all; lower in red and processed meat, low in sugar-sweetened foods and drinks and refined grains.	Addition of “foods appropriate for personal medical conditions”	“[It] depends on personal health. I cannot eat whole grains because of the potassium.”
		Addition of food item examples in multiple categories	“Is pork considered a red meat?”
	*Physical opportunity description:* What is PHYSICAL opportunity? Your surroundings (e.g., the places where you live, work, and visit) provide the opportunity to practice healthy eating. Physical opportunity also includes material and non-material resources like money, equipment, time, and transportation.	Added emphasis on “access” and providing nutrition-related examples of resources (kitchen equipment and appliances)	“[I’m thinking] more along the lines of physical activity, not opportunity. What if you said ‘physical access’?”
	*Social opportunity description:* What is SOCIAL opportunity? Influences from other people, social cues, and cultural norms provide the opportunity to practice healthy eating. (e.g., Support from friends and family)	Clarification of the meaning of cultural norms related to healthy eating behaviors	“[I’m] struggling with cultural norms. What is meant by culture?”
	*Social opportunity item:* Healthy eating is common for people in my culture.	Modification to emphasize aspect of social connectedness	“How do I define my culture? I have trouble with cultural norms. People’s definitions will be different for culture.”
			“Maybe try saying, ‘Healthy eating is common is my social circles.’“
	*Reflective motivation item:* The benefits of healthy eating outweigh the costs.	Modification to use “positives” and “negatives” rather than “benefits” and “costs”	“When I hear ‘costs’ I think of money, but I know there are other costs to healthy eating. But by seeing the word, it almost makes me focus only on the money cost.”
	*Physical capability item:* My body feels fully able to allow me to practice healthy eating.	Clarification of “fully able” and addition of examples	“Could you include something more descriptive? Like, ‘I am able to go to the grocery store, purchase the food, prepare the food, store it, able to chew it, and clean up.’“
Items achieving moderate understanding (*n* = 7)	*Social opportunity item:* My friends and family are supportive of my healthy eating practices.	Separation of the double-barreled question into two separate items	“In some cases maybe your friends are pushing you in one direction with food and then your family in another. Maybe split the question into two.”
	*Reflective motivation description:* What is REFLECTIVE motivation? Having goals, making decisions, and conscious planning and beliefs about the good and bad consequences of healthy eating. (e.g., I intend to…; I have the desire to…; I feel the need to practice healthy eating)	Clarification of “good and bad consequences of healthy eating”	“Do I consider why I desire to make certain healthy or non-healthy eating patterns?”
	*Reflective motivation item:* I want to practice healthy eating to improve my overall health.	Addition of the word “maintain”	“I think maintain would be better than improve… I suppose I could always improve but I am more focused on maintaining [my health].”
	*Reflective motivation item:* I think that I should practice healthy eating so that I can lower my risks related to chronic disease.	Addition of examples of chronic diseases	“The things that come to mind with chronic diseases are diabetes, heart issues, arthritis. Any chronic disease that can be impacted by healthy eating.”
	*Automatic motivation description:* What is AUTOMATIC motivation? Doing something without needing to think about it or having to consciously remember. (e.g., Healthy eating is something I do before I realize I’m doing it.)	Inclusion of the word “habit”	“What you are used to—your habits.”
	*Physical capability description:* What is PHYSICAL capability? Having the physical skill, strength, or stamina needed to practice healthy eating. (e.g., I have enough physical strength and energy, I can overcome physical limitations, I have the necessary physical skills)	Modification to clarify meaning of “overcome any physical limitations”	“The example part is confusing. what does ‘I can overcome physical limitations’ mean?”
	*Physical capability item:* I can overcome any physical limitations (e.g., illness, disease, disability) to practice healthy eating.	Modification to clarify meaning of “overcome any physical limitations”	“I’m still struggling with ‘overcome’. I would suggest: ‘I do not have any physical limitations that would keep me from practicing healthy eating.’”

Participants appeared to have the greatest difficulty understanding the descriptions of each subscale (5 out of 6 descriptions required revision). In terms of achieving understanding of items within subscales, participants appeared to have the most difficulty with items in the reflective motivation subscale (3 out of 7 items required modification). Participants expressed the greatest levels of understanding for items in the psychological capability, automatic motivation, and physical opportunity subscales, in which there were zero item revisions required. These results indicate adequate face validity of instrument items among the target population. After making revisions to problematic items and descriptions, 34 items across six subscales were pilot tested in Phase II.

### 3.3 Phase II: pilot-testing

#### 3.3.1 Descriptive statistics

Descriptive statistics for capability, opportunity, motivation, and REAP-S score are presented in Table 4. There were no significant differences in capability, opportunity, motivation, or REAP-S score based on age, gender, education, or employment status.

**Table 4:** Descriptive statistics for capability, opportunity, motivation, and REAP-S scores.

	Descriptive statistics (*n* = 81)					
	Minimum	Maximum	Mean	SD	Skewness	Std. Error
Capability^a^	1.00	5.00	4.71	0.83	−3.99	0.27
Opportunity^a^	2.00	5.00	4.45	0.52	−2.26	0.27
Motivation^a^	2.25	5.00	4.05	0.61	−0.67	0.27
REAP-S score^b^	21.00	38.00	31.74	3.87	−0.60	0.27

a Possible score ranging from 1 to 5, with higher score indicating higher levels of the variable.

b Possible score ranging from 13 to 39, with higher scores indicating higher dietary quality.

#### 3.3.2 Acceptability analysis

Each subscale on the new instrument concluded with three items to assess the acceptability of the subscale, using sliding scales ranging from 0 to 10, to measure ease of reading and understanding of the subscales. Ratings for ease of reading were all very high, ranging from the lowest mean for physical capability (*M* = 9.2 ± 1.0) to the highest mean for physical opportunity (*M* = 9.5 ± 0.8). These scores indicated that the items were all very easy to read among this well-educated sample. Ratings for understanding were similarly high, with the lowest mean occurring for psychological capability (*M* = 9.4 ± 0.9), to the highest mean occurring for physical opportunity (*M* = 9.6 ± 0.7), indicating high levels of understanding of the items across all six subscales. The open-ended comments at the end of each subscale were coded into positive, negative, or neutral categories to help gauge the acceptability of the new instrument. A summary of acceptability measures from Phase II is displayed in Table 1. The number of participants providing any comment ranged from eight (physical opportunity) to 15 (reflective motivation) per subscale. The majority of participants did not provide any comments on these open-ended questions. The most prominent theme across all six subscales was the dislike/confusion regarding the reverse scored item in each subscale (e.g., “I do NOT feel that I have the social opportunity for healthy eating.”), followed by technical difficulties in using the online survey platform. Other comments tended to clarify the participants’ reasoning behind their selected answers. After considering the results of internal reliability tests and content analysis, the subscales were modified to omit the final question that was reverse scored in each subscale.

Finally, extreme ceiling effects were evident for 27 out of 28 final (see description below of reduction from 34 to 28) items when examined separately. The one item that did not reach a ceiling effect was, “My healthy eating practices tend to happen mindlessly,” where only 9 participants selected the highest score for the item. However, when the items were loaded into their six respective subscales and examined for ceiling or floor effects, just four subscales had ceiling effects present. Since there were no items or subscales with floor effects, only the ceiling effects are presented in Table 5.

**Table 5:** Ceiling effects for six subscales.

	Frequency of achieving highest possible score (*n* = 81)		
Subscale	N	%	Ceiling effect present?*
Physical capability	63	77.8	✓
Psychological capability	50	61.7	✓
Reflective motivation	19	23.5	✓
Automatic motivation	4	4.9	
Physical opportunity	59	72.8	✓
Social opportunity	11	13.6	

*Ceiling effects are present when >15% of participants achieve the highest possible score on a given subscale.

#### 3.3.3 Reliability analysis

The COM-HE instrument achieved acceptable internal consistency as measured by Cronbach’s alpha levels. These results are presented in Table 6. Including reverse-scored items (34 item version), subscale Cronbach’s alphas ranged from 0.826 to 0.915, with a total alpha of 0.717. After removing these items, internal consistency improved for most subscales, and qualitative feedback also indicated participant dislike of reverse-wording. Based on these findings and supporting literature suggesting issues with negatively-worded items (30), reverse-scored items were omitted from further analyses (leaving 28 items). All subscales then showed acceptable reliability based on the target Cronbach’s alpha range of 0.7–0.95 (15), though very high alphas for the physical and psychological capability subscales suggest potential item redundancy. Forty-four participants completed the COM-HE instrument at two different timepoints, within 14 days of one another, to determine test–retest reliability. The ICCs ranged from 0.141 (psychological capability) to 0.854 (physical capability), with five of the six subscales indicating moderate–good test–retest reliability.

**Table 6:** Scale reliability.

	Pre-modification		Post-modification		Post-modification test–retest reliability
Subscale	Number of items	Cronbach’s alpha	Number of items	Cronbach’s alpha	ICC
Physical capability	5	0.851	4	0.986	0.854
Psychological capability	6	0.873	5	0.938	0.141
Reflective motivation	7	0.915	6	0.898	0.595
Automatic motivation	5	0.841	4	0.847	0.595
Physical opportunity	5	0.826	4	0.871	0.500
Social opportunity	6	0.899	5	0.888	0.810

Post-modification scores reflected items remaining following removal of the reverse-scored items from the 34-item scale. The modified scale included 28 items.

#### 3.3.4 Validity analysis

Results for construct validity revealed mixed results for uni-dimensionality. Paired component loadings to extract two principal components (PCs) were conducted for C, O, and M, and these results are presented in Table 7. when all 28 COM-HE items were loaded to extract six principal components (PCs), the Kaiser-Meyer-Olkin (KMO) value was 0.815, suggesting adequate sampling to conduct exploratory factor analysis (26). Six PCs explained 81.8% of the variance, but only 5 of these had eigenvalues >1, suggesting that the COM-HE instrument may instead be a five-factor model explaining 78.77% of the variance. This indicates potential crossover between the six COM-HE subscales rather than true uni-dimensionality of each.

**Table 7:** Paired principal component analyses.

	Capability: total variance explained					
	Initial eigenvalues			Extraction sums of squared loadings		
Component	Total	% of variance	Cumulative %	Total	% of variance	Cumulative %
1	7.606	84.509	84.509	7.606	84.509	84.509
2	0.830	9.224	93.733	0.830	9.224	93.733
3	0.195	2.165	95.899			
4	0.136	1.516	97.414			
5	0.087	0.968	98.383			
6	0.069	0.768	99.151			
7	0.038	0.426	99.577			
8	0.024	0.261	99.838			
9	0.015	0.162	100.000			
Extraction method: Principal component analysis						
KMO = 0.896 (*p* < 0.001).						

	Opportunity: total variance explained					
	Initial eigenvalues			Extraction sums of squared loadings		
Component	Total	% of variance	Cumulative %	Total	% of variance	Cumulative %
1	4.151	46.123	46.123	4.151	46.123	46.123
2	2.434	27.049	73.172	2.434	27.049	73.172
3	0.661	7.341	80.513			
4	0.456	5.070	85.583			
5	0.397	4.410	89.992			
6	0.362	4.028	94.020			
7	0.246	2.737	96.757			
8	0.163	1.814	98.571			
9	0.129	1.429	100.000			
Extraction method: principal component analysis						
KMO = 0.786 (*p* < 0.001).						

	Motivation: total variance explained					
	Initial eigenvalues			Extraction sums of squared loadings		
Component	Total	% of variance	Cumulative %	Total	% of variance	Cumulative %
1	4.404	44.040	44.040	4.404	44.040	44.040
2	2.665	26.650	70.690	2.665	26.650	70.690
3	0.831	8.314	79.005			
4	0.626	6.264	85.268			
5	0.478	4.785	90.053			
6	0.294	2.937	92.990			
7	0.225	2.249	95.239			
8	0.192	1.924	97.164			
9	0.165	1.652	98.815			
10	0.118	1.185	100.000			
Extraction method: principal component analysis						
KMO = 0.800 (*p* < 0.001).						

Discriminant and convergent validity were also examined to measure construct validity using Spearman correlations expressed by Spearman’s rho (r) between the six subscales. These results are presented in Table 8. Physical capability and psychological capability had a moderate positive correlation, reflective motivation and automatic motivation had a weak positive correlation, and physical opportunity and social opportunity were not significantly correlated. These results indicate convergent validity within subscales, although only at a moderate level. Significant correlations were also seen between several sets of subscales, with moderate correlations between automatic motivation and social opportunity, reflective motivation and social opportunity, social opportunity and psychological capability, physical opportunity and psychological capability, and a small correlation between reflective motivation and psychological capability. Some significant positive correlations remained when the subscales were combined into their respective C, O, and M scales. Opportunity and motivation were moderately positively correlated, capability and opportunity were weakly positively correlated, and motivation and opportunity were not significantly correlated. These results reinforce the interactions between COM-B components (Table 9).

**Table 8:** Discriminant and convergent validity by subscales.

Spearman’s rho (ρ)	Physical capability	Psychological capability	Reflective motivation	Automatic motivation	Physical opportunity	Social opportunity
Physical capability	1.000	0.537**	0.112	−0.001	0.201	0.210
Psychological capability	0.537**	1.000	0.264*	0.027	0.305**	0.352**
Reflective motivation	0.112	0.264*	1.000	0.368**	0.213	0.391**
Automatic motivation	−0.001	0.027	0.368**	1.000	0.012	0.511**
Physical opportunity	0.201	0.305**	0.213	0.012	1.000	0.181
Social opportunity	0.210	0.352**	0.391**	0.511**	0.181	1.000

**Correlation is significant at the 0.01 level (2-tailed). *Correlation is significant at the 0.05 level (2-tailed).

**Table 9:** Discriminant and convergent validity by C, O, M scales.

Spearman’s rho (ρ)	Capability	Opportunity	Motivation
Capability	1.000	0.422**	0.118
Opportunity	0.422**	1.000	0.553**
Motivation	0.118	0.553**	1.000

**Correlation is significant at the 0.01 level (2-tailed).

Multiple regression analyses were conducted to assess the associations between the six COM-HE subscales and REAP-S scores among community-dwelling older adults. These results are presented in Table 10. When assessed together, the six COM subscales had a moderate positive association with REAP-S scores, explaining 9.9% of the variance. Motivation had a positive moderate association with REAP-S scores. There was also a weak positive correlation between opportunity and REAP-S scores, and capability did not have a significant association with REAP-S scores among community-dwelling older adults.

**Table 10:** Concurrent validity between COM-HE and REAP-S scores.

Criterion	*r*	*R* ^2^	Adjusted *R*^2^	*p*-value
Capability	0.069	0.005	−0.008	0.542
Physical capability	0.060	0.004	−0.009	0.595
Psychological capability	0.076	0.006	−0.007	0.502
Opportunity	0.247*	0.061	0.049	0.026
Physical opportunity	0.151	0.023	0.010	0.178
Social opportunity	0.233*	0.054	0.042	0.036
Motivation	0.379**	0.144	0.133	<0.001
Reflective motivation	0.189	0.036	0.024	0.090
Automatic motivation	0.370**	0.137	0.126	<0.001
Six COM subscales together	0.409*	0.167	0.099	0.031

*Correlation is significant at *p* < 0.05. **Correlation is significant at *p* < 0.001.

## 4 Discussion

### 4.1 Main findings

The current study provides a detailed description of the development and application of a survey instrument developed to measure the perceived capability, opportunity, and motivation for healthy eating behavior among community-dwelling older adults. To our knowledge, this is the first study to generate and evaluate original questionnaire items related to healthy eating behaviors among the older adult population based upon the COM-B model (10). There were five main findings of this mixed methods study.

First, the COM-HE instrument is well accepted by the target population, with both qualitative and quantitative analyses indicating high levels of acceptability. An interesting acceptability finding was that while negative comments did not comprise a large proportion of the comments overall, the physical and psychological capability subscales had larger proportions of negative comments as compared to the other four subscales. This may have been due in part to the two capability subscales having been arranged first in the online questionnaire, and the phenomenon of survey fatigue which involves lower levels of effort as a survey progresses and is also observed more often with open-ended types of questions (14). Although the acceptability results may have been influenced by the study sample of mostly well-educated older adults, the results provide a good starting point for assessing the overall appropriateness of the new instrument and can be used to guide future iterations of the instrument. It is also worthwhile to note that while the original COM-B instrument contained only six items (10) and provided a sufficient foundation for instrument development in the present study, one item for each COM subscale is not sufficient to measure the multiple concepts that each subscale includes. For example, our definition of physical opportunity spans across multiple features of the subdomain including proximity to healthy food sources, financial resources, transportation, kitchen equipment, and time—it would be ill-advised to attempt to measure these distinct aspects of physical opportunity with a single item on a questionnaire. To this end, it was deduced that the inclusion of up to six items per subscale did not attenuate the ease of reading nor the perceived understanding of the COM-HE instrument among this sample of community-dwelling older adults.

The second key finding provided evidence for reliability when considering internal consistency of the COM-HE instrument. Cronbach’s alphas for each of the six subscales separately were greater than 0.7, indicating satisfactory internal consistency (27). These alpha levels show that each subscale contains items that are adequately interrelated. However, the alpha levels reaching >0.9 in two subscales (physical and psychological capability) may indicate redundancy of some items, suggesting that these two subscales may benefit from the omission of very similar items or the addition of items that measure a different aspect of the respective subscales in future versions of the instrument. The internal consistency in the present study were similar to that of a questionnaire which sourced previously-validated measures from the Theoretical Domains Framework and mapped them onto the COM-B domains to measure eating and physical activity behaviors among young adults (31). The internal consistency results for the COM-HE instrument were slightly higher than a questionnaire developed to measure the capability, opportunity, and motivation for exercise among children with obesity (32). Overall, the COM-HE instrument achieved good internal consistency and can be considered reliable among well-educated community-dwelling older adults.

Third, the instrument displays varying levels of construct validity based on PCA and Spearman correlations. PCA revealed a five-component model, indicating that the six COM-HE subscales may not map evenly onto the components that were extracted. When the subscales were paired according to C, O, and M, both opportunity and motivation each had two-component models, but capability resulted in a one-component model. This implies that the two capability subscales may only measure one component. These PCA results are particularly interesting when considering that the psychological and physical capability subscales also had the highest internal consistency reliability, indicating potential redundancy within each subscale. When taken together, these findings could signify that the two capability subscales require additional modification to further differentiate between physical and psychological capability.

Fourth, the findings for discriminant and convergent validity contributed to better understanding the associations within and between domains of the COM-B model. The two capability subscales were moderately positively correlated, the two motivation subscales were weakly positively correlated, and the two opportunity subscales were not significantly correlated with one another. However, when the subscales were combined and evaluated against one another, the opportunity scale was significantly positively correlated with capability and motivation. These findings highlight the interrelatedness of COM components, where all three domains act directly and indirectly to influence behavior, and are similar to those of a study evaluating the correlations between COM factors for preventative care provided by veterinarians (33). Overall, the literature is limited in terms of evaluating correlations between COM factors for human health behaviors, indicating a need for further investigation.

The fifth key finding is that the six COM subscales were moderately positively correlated with self-reported dietary quality. The six correlates (physical and psychological capability, reflective and automatic motivation, and physical and social opportunity) explained 9.9% of the variance in REAP-S scores, which is not inconsequential considering the complex nature of internal and external factors that influence dietary quality. However, the explained variance is less than what has been observed for the COM-B model with eating behavior among young adults with a mean age of 24.9 years, where COM-B explained 23% of variance in eating behavior (31). However, the variance in the current study is comparable to the variance explained by other behavioral theories such as the theory of reasoned action, which has been found to explain 6.25–30.25% of the variance in nutrition-related behaviors (34), and the social cognitive theory, which has been found to explain 14–61% of variance in dietary intake (35). In the present study, motivation had the strongest positive correlation with dietary quality out of the three COM scales, which is also similar to the aforementioned study where motivation explained 23% of the variance in eating behaviors for young adults. Furthermore, the observed positive association between social opportunity and healthy eating behaviors in the present study contrasts with the findings of a qualitative study in which most independently-living older adults indicated that they did not pay much attention to, and their behaviors were not influenced by, the eating habits of other people (36). Lastly, capability added negligible contributions to this measure of concurrent validity, which again suggests that either (1) capability is not correlated with dietary quality among this sample of older adults, or (2) the two capability subscales did not adequately measure what they were intended to measure; or perhaps a combination of both, considering the influence of ceiling effects. Overall, the instrument displays moderate validity when assessing concurrent associations between the COM domains and dietary quality, especially within the opportunity and motivation scales.

### 4.2 Strengths and limitations

A main strength of the study was the mixed methods approach to developing the COM-HE instrument. Qualitative and quantitative methods each have their own strengths and limitations, whereas combining both methods can address both the subjective (i.e., perceptions, life experiences) and objective (i.e., health outcomes) aspects of a research question (37). The newly generated items in the COM-HE instrument align well with correlates of healthy eating behavior among community-dwelling older adults which have been previously described in the literature (36, 38, 39). We considered a breadth of intrapersonal, interpersonal, and environmental correlates of eating behaviors and included multiple factors within each subscale definition. This development of multifaceted subscales, rather than using a single item to measure a broad subdomain, is a major strength considering the complex interactions between capability, opportunity, and motivation for healthy eating behaviors. Another important strength of the COM-HE instrument is its potential for direct applicability in real-world settings, providing a practical tool for researchers, policymakers, and healthcare professionals to assess behavioral determinants of healthy eating in older adults. However, it is important to note that the COM-HE instrument may benefit from further refinement, particularly for the capability subscales. By identifying specific barriers and facilitators across the COM-B components, the instrument can guide the development of targeted behavioral nutrition interventions and inform evidence-based public health strategies. At this stage, the COM-HE is best placed to provide a starting point for researchers for modifying and adapting for further testing among different populations and contexts. Future research should determine whether the instrument may have clinical relevance, aiding clinicians and dietitians in tailoring individualized nutrition counseling and behavior change strategies.

Careful attention should be paid to the items in the physical and psychological capability subscales should the instrument undergo further revision and testing. This is particularly true given the test-reliability results. Of note, the psychological capability subscale showed poor test–retest reliability, and it may be the case that scores changed in response to participant reflections on the COM-HE instrument content, situational factors at the time of each test administration, or instability in the construct itself. Future research should consider the timing of COM-HE administration within study designs.

The most obvious limitation with the current study was the homogeneous participant sample in terms of race and education level. With 100% of participants (*n* = 81) reporting they were non-Hispanic White, and a large majority holding a bachelor’s degree or higher, it is highly probable that these characteristics were significant contributors to the observed ceiling effects and may have influenced the relatively high dietary quality scores using the REAP-S tool. This homogeneity may limit the generalizability of the findings to more diverse populations, where cultural, socioeconomic, and environmental factors could influence healthy eating behaviors differently. The highly educated individuals in the sample may have also influenced the acceptability results that tended toward very high levels of understanding and ease of reading the instrument. Additionally, cultural factors may influence how individuals perceive and respond to items related to automatic motivation, such as ingrained habits, emotional responses, and social norms surrounding food and eating behaviors. Given that our sample lacked ethnic and cultural diversity, the current findings may not fully capture how cultural beliefs and practices impact automatic motivational drivers of healthy eating in more diverse populations. Another limitation is the absence of data regarding income levels, marital status, living arrangement, and chronic disease status. These would have been helpful factors to make possible distinctions from the otherwise homogeneous study sample, but we aimed to keep the participant burden low by controlling the number of demographic-related items on the questionnaire. Future research should aim to validate the COM-HE instrument in more ethnically diverse populations to ensure its applicability across varying demographic contexts.

## 5 Conclusion

Findings from this mixed methods study provide insight into the capability, opportunity, and motivation for healthy eating behaviors among well-educated community-dwelling older adults. Overall, the COM-HE instrument was acceptable, internally consistent, reliable (with the possible exception of psychological capability), and moderately valid for identifying important healthy eating correlates. When assessed simultaneously, all six subscales were correlated with dietary quality, and motivation and opportunity were identified as having the strongest positive correlations with dietary quality when assessed separately. This study adds to the literature, an expanded version of the original general six-item COM-B questionnaire (10), with an emphasis on healthy eating behaviors among community-dwelling older adults, a rapidly growing segment of the U.S. and global population. Future research should replicate, modify, and validate the COM-HE instrument in ethnically and culturally diverse populations to ensure its applicability across different demographic contexts and further examine its predictive validity in relation to dietary outcomes.

## Data Availability

The raw data supporting the conclusions of this article will be made available by the authors, without undue reservation.
