# Supplementary material for: Evaluating correlates of healthy eating and dietary quality among older adults: a mixed methods approach to development and application of a new survey instrument
Source: Front Public Health. 2025 Oct 20;13:1661573. doi: 10.3389/fpubh.2025.1661573 (PMC12581219; doi:10.3389/fpubh.2025.1661573)
Supplement: Supplementary file 1 [file Table_1.DOCX]

Supplementary Material

###### COM-HE Instrument

A copy of the COM-HE instrument as it was administered during pilot testing

The Capability, Opportunity, and Motivation for Healthy Eating (COM-HE) Instrument

**Please take your time to read this introduction carefully.**
This survey will ask questions about the opportunities, motivations, and capabilities that influence what and how you eat.
 
Health is more than just the absence of disease. The definition of “healthy eating” used in this survey involves several aspects of health. When a question mentions “healthy eating,” it means choosing a variety of foods and meals that will improve health. 
 
These foods and meals are well-balanced and eaten in adequate amounts to support physical, social, and mental well-being. Healthy eating also includes some of the actions that make it possible to eat healthy foods. Some examples include being able to get healthy foods and having the knowledge, skills, and physical ability to prepare healthy foods. 
 
This definition of healthy eating recognizes that there is more to healthy eating than the type and amount of foods eaten. It also involves practicing mindfulness by slowing down and paying attention to the food being eaten. Healthy eating includes having a healthy relationship with food. It recognizes how food can make people feel socially and culturally connected to one another. Healthy eating takes into account your personal medical conditions and the foods you should eat or avoid to stay well.
  **A diet that reflects healthy eating includes:**
• Vegetables
• Fruits
• Whole grains (e.g. 100% whole wheat bread, oatmeal, whole wheat tortilla, etc.)
• Low-fat dairy or dairy alternatives
• Seafood, beans/legumes, nuts, lean meats and poultry
• Moderate consumption of alcohol (up to 2 drinks per day for men; up to 1 drink per day for women) if alcohol is consumed at all
• Lower in red and processed meat (e.g. bacon, deli meats, hot dogs, etc.)
• Low in sugar-sweetened foods and drinks and refined grains (e.g. white breads, desserts, sugary cereals, etc.)
 
Healthy eating does **not** include trying the latest diet, restricting entire food groups for non-medical reasons, or eating excess added sugars, saturated fats, and *trans* fats.

End of Block: Introduction

Start of Block: Physical Capability

**What is PHYSICAL capability?**
 
Having the physical skill, strength, or stamina needed to practice healthy eating.
 
(e.g., I have enough physical strength and energy to obtain healthy foods, I can work around any physical limitations that make it difficult to prepare healthy food, I have the necessary physical skills to practice healthy eating)
 
**Remember:** HEALTHY EATING supports physical, social, and mental well-being. Healthy eating: includes a variety of vegetables, fruits, whole grains, low-fat dairy or dairy alternative, seafood, legumes, and nuts; is moderate in alcohol; is lower in red and processed meat; and is low in sugar-sweetened foods and drinks and refined grains.

Q1
I have the PHYSICAL capability needed to practice healthy eating.

- Strongly disagree
- Somewhat disagree
- Neither agree nor disagree
- Somewhat agree
- Strongly agree

Q2 I feel that my body is fully capable of doing everything that is needed to practice healthy eating (e.g. obtaining healthy foods, prepping and cooking, chewing and swallowing, etc.)

- Strongly disagree
- Somewhat disagree
- Neither agree nor disagree
- Somewhat agree
- Strongly agree

Q3 For the most part, I can find a way to work around physical limitations (e.g. illness, disease, disability) to practice healthy eating.

- Strongly disagree
- Somewhat disagree
- Neither agree nor disagree
- Somewhat agree
- Strongly agree

Q4 On a day-to-day basis, I can practice healthy eating without any assistance.

- Strongly disagree
- Somewhat disagree
- Neither agree nor disagree
- Somewhat agree
- Strongly agree

Q5 I do NOT feel that I have the physical capability to practice healthy eating.

- Strongly disagree
- Somewhat disagree
- Neither agree nor disagree
- Somewhat agree
- Strongly agree

Overall, did you find the previous set of 5 questions difficult or easy to read?

|  | Difficult to read | Easy to read |
| --- | --- | --- |

|  | 0 | 1 | 2 | 3 | 4 | 5 | 6 | 7 | 8 | 9 | 10 |
| --- | --- | --- | --- | --- | --- | --- | --- | --- | --- | --- | --- |

|  | 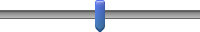 |
| --- | --- |

Overall, how confident are you that you understood what the previous set of 5 questions were asking?

|  | Not at all confident | Very confident |
| --- | --- | --- |

|  | 0 | 1 | 2 | 3 | 4 | 5 | 6 | 7 | 8 | 9 | 10 |
| --- | --- | --- | --- | --- | --- | --- | --- | --- | --- | --- | --- |

|  | 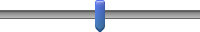 |
| --- | --- |

(Optional) Comments on any of the previous set of 5 questions?

________________________________________________________________

End of Block: Physical Capability

Start of Block: Psychological Capability

**What is PSYCHOLOGICAL capability?**


Knowledge, comprehension, and reasoning needed to practice healthy eating.


(e.g., Having the necessary know-how, understanding, and ability to figure things out; Being able to engage in tasks requiring memory, attention and decision-making processes).


**Remember:** HEALTHY EATING supports physical, social, and mental well-being. Healthy eating: includes a variety of vegetables, fruits, whole grains, low-fat dairy or dairy alternative, seafood, legumes, and nuts; is moderate in alcohol; is lower in red and processed meat; and is low in sugar-sweetened foods and drinks and refined grains.

Q6 I am PSYCHOLOGICALLY capable of practicing healthy eating.

- Strongly disagree
- Somewhat disagree
- Neither agree nor disagree
- Somewhat agree
- Strongly agree

Q7 I can describe the actions that I should take to practice healthy eating.

- Strongly disagree
- Somewhat disagree
- Neither agree nor disagree
- Somewhat agree
- Strongly agree

Q8 I know how to apply the idea of healthy eating to my own life.

- Strongly disagree
- Somewhat disagree
- Neither agree nor disagree
- Somewhat agree
- Strongly agree

| Page Break |  |
| --- | --- |

Q9 I have the clear thinking that I need for all aspects of healthy eating.

- Strongly disagree
- Somewhat disagree
- Neither agree nor disagree
- Somewhat agree
- Strongly agree

Q10 My abilities to understand and remember are helpful for practicing healthy eating.

- Strongly disagree
- Somewhat disagree
- Neither agree nor disagree
- Somewhat agree
- Strongly agree

Q11 I do NOT think that I am psychologically capable of practicing healthy eating.

- Strongly disagree
- Somewhat disagree
- Neither agree nor disagree
- Somewhat agree
- Strongly agree

| Page Break |  |
| --- | --- |

Overall, did you find the previous set of 6 questions difficult or easy to read?

|  | Difficult to read | Easy to read |
| --- | --- | --- |

|  | 0 | 1 | 2 | 3 | 4 | 5 | 6 | 7 | 8 | 9 | 10 |
| --- | --- | --- | --- | --- | --- | --- | --- | --- | --- | --- | --- |

|  | 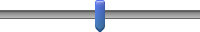 |
| --- | --- |

Overall, how confident are you that you understood what the previous set of 6 questions were asking?

|  | Not at all confident | Very confident |
| --- | --- | --- |

|  | 0 | 1 | 2 | 3 | 4 | 5 | 6 | 7 | 8 | 9 | 10 |
| --- | --- | --- | --- | --- | --- | --- | --- | --- | --- | --- | --- |

|  | 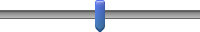 |
| --- | --- |

(Optional) Comments on any of the previous set of 6 questions?

________________________________________________________________

End of Block: Psychological Capability

Start of Block: Reflective Motivation

**What is REFLECTIVE motivation?**


Having goals, making decisions, and consciously planning to practice healthy eating. Reflective motivation involves intentionally making an effort to practice healthy eating after considering the positives and negatives of choosing to eat healthfully.


(e.g., I intend to…; I have the desire to…; I feel the need to practice healthy eating)


**Remember:** HEALTHY EATING supports physical, social, and mental well-being. Healthy eating: includes a variety of vegetables, fruits, whole grains, low-fat dairy or dairy alternative, seafood, legumes, and nuts; is moderate in alcohol; is lower in red and processed meat; and is low in sugar-sweetened foods and drinks and refined grains.

Q12 I am motivated to practice healthy eating.

- Strongly disagree
- Somewhat disagree
- Neither agree nor disagree
- Somewhat agree
- Strongly agree

Q13 I want to practice healthy eating to maintain or improve my overall health.

- Strongly disagree
- Somewhat disagree
- Neither agree nor disagree
- Somewhat agree
- Strongly agree

Q14 I think I should practice healthy eating so that I can lower my risks related to chronic disease (e.g. heart disease, cancer, diabetes, etc.).

- Strongly disagree
- Somewhat disagree
- Neither agree nor disagree
- Somewhat agree
- Strongly agree

Q15 On a day-to-day basis, I intentionally practice healthy eating.

- Strongly disagree
- Somewhat disagree
- Neither agree nor disagree
- Somewhat agree
- Strongly agree

Q16 The positives that come from healthy eating outweigh the negatives.

- Strongly disagree
- Somewhat disagree
- Neither agree nor disagree
- Somewhat agree
- Strongly agree

Q17 If I practice healthy eating, I expect that I will experience many health benefits (e.g. more energy, reduced risk of illness, living longer).

- Strongly disagree
- Somewhat disagree
- Neither agree nor disagree
- Somewhat agree
- Strongly agree

Q18 I do NOT want to practice healthy eating.

- Strongly disagree
- Somewhat disagree
- Neither agree nor disagree
- Somewhat agree
- Strongly agree

| Page Break |  |
| --- | --- |

Overall, did you find the previous set of 7 questions difficult or easy to read?

|  | Difficult to read | Easy to read |
| --- | --- | --- |

|  | 0 | 1 | 2 | 3 | 4 | 5 | 6 | 7 | 8 | 9 | 10 |
| --- | --- | --- | --- | --- | --- | --- | --- | --- | --- | --- | --- |

|  | 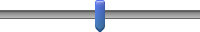 |
| --- | --- |

Overall, how confident are you that you understood what the previous set of 7 questions were asking?

|  | Not at all confident | Very confident |
| --- | --- | --- |

|  | 0 | 1 | 2 | 3 | 4 | 5 | 6 | 7 | 8 | 9 | 10 |
| --- | --- | --- | --- | --- | --- | --- | --- | --- | --- | --- | --- |

|  | 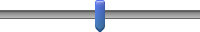 |
| --- | --- |

(Optional) Comments on any of the previous set of 7 questions?

________________________________________________________________

End of Block: Reflective Motivation

Start of Block: Automatic Motivation

**What is AUTOMATIC motivation?**


Doing something without needing to think about it or having to consciously remember. Automatic motivation refers to something that is a habit.


(e.g., Healthy eating is something I do before I realize I’m doing it.)


**Remember:** HEALTHY EATING supports physical, social, and mental well-being. Healthy eating: includes a variety of vegetables, fruits, whole grains, low-fat dairy or dairy alternative, seafood, legumes, and nuts; is moderate in alcohol; is lower in red and processed meat; and is low in sugar-sweetened foods and drinks and refined grains.

Q19 Healthy eating is something I do automatically.

- Strongly disagree
- Somewhat disagree
- Neither agree nor disagree
- Somewhat agree
- Strongly agree

Q20 Healthy eating comes naturally to me.

- Strongly disagree
- Somewhat disagree
- Neither agree nor disagree
- Somewhat agree
- Strongly agree

Q21 Healthy eating is more of a habit than something I need to remember consciously.

- Strongly disagree
- Somewhat disagree
- Neither agree nor disagree
- Somewhat agree
- Strongly agree

Q22 My healthy eating practices tend to happen mindlessly.

- Strongly disagree
- Somewhat disagree
- Neither agree nor disagree
- Somewhat agree
- Strongly agree

Q23 For me to practice healthy eating, I really have to remember and plan for it.

- Strongly disagree
- Somewhat disagree
- Neither agree nor disagree
- Somewhat agree
- Strongly agree

|  |
| --- |

Overall, did you find the previous set of 5 questions difficult or easy to read?

|  | Difficult to read | Easy to read |
| --- | --- | --- |

|  | 0 | 1 | 2 | 3 | 4 | 5 | 6 | 7 | 8 | 9 | 10 |
| --- | --- | --- | --- | --- | --- | --- | --- | --- | --- | --- | --- |

|  | 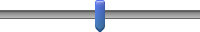 |
| --- | --- |

Overall, how confident are you that you understood what the previous set of 5 questions were asking?

|  | Not at all confident | Very confident |
| --- | --- | --- |

|  | 0 | 1 | 2 | 3 | 4 | 5 | 6 | 7 | 8 | 9 | 10 |
| --- | --- | --- | --- | --- | --- | --- | --- | --- | --- | --- | --- |

|  | 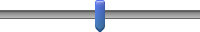 |
| --- | --- |

(Optional) Comments on any of the previous set of 5 questions?

________________________________________________________________

End of Block: Automatic Motivation

Start of Block: Physical Opportunity

| Page Break |  |
| --- | --- |

**What is PHYSICAL opportunity?**


Your surroundings (e.g., the places where you live, work, and visit) give you a chance to practice healthy eating.


Physical opportunity also includes having access to material and non-material resources like money, kitchen equipment and appliances, time, and transportation. Physical opportunity refers to having the right resources available to help you practice healthy eating.


**Remember:** HEALTHY EATING supports physical, social, and mental well-being. Healthy eating: includes a variety of vegetables, fruits, whole grains, low-fat dairy or dairy alternative, seafood, legumes, and nuts; is moderate in alcohol; is lower in red and processed meat; and is low in sugar-sweetened foods and drinks and refined grains.

Q24 I have the PHYSICAL opportunity that I need for healthy eating.

- Strongly disagree
- Somewhat disagree
- Neither agree nor disagree
- Somewhat agree
- Strongly agree

Q25 The right foods are available to me for healthy eating.

- Strongly disagree
- Somewhat disagree
- Neither agree nor disagree
- Somewhat agree
- Strongly agree

Q26 I have the resources (e.g. time, money, transportation, kitchen equipment) that I need for healthy eating on a day-to-day basis.

- Strongly disagree
- Somewhat disagree
- Neither agree nor disagree
- Somewhat agree
- Strongly agree

| Page Break |  |
| --- | --- |

Q27 I have access to what I need for healthy eating.

- Strongly disagree
- Somewhat disagree
- Neither agree nor disagree
- Somewhat agree
- Strongly agree

Q28 I do NOT feel that I have the physical opportunity for healthy eating.

- Strongly disagree
- Somewhat disagree
- Neither agree nor disagree
- Somewhat agree
- Strongly agree

| Page Break |  |
| --- | --- |

Overall, did you find the previous set of 5 questions difficult or easy to read?

|  | Difficult to read | Easy to read |
| --- | --- | --- |

|  | 0 | 1 | 2 | 3 | 4 | 5 | 6 | 7 | 8 | 9 | 10 |
| --- | --- | --- | --- | --- | --- | --- | --- | --- | --- | --- | --- |

|  | 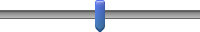 |
| --- | --- |

Overall, how confident are you that you understood what the previous set of 5 questions were asking?

|  | Not at all confident | Very confident |
| --- | --- | --- |

|  | 0 | 1 | 2 | 3 | 4 | 5 | 6 | 7 | 8 | 9 | 10 |
| --- | --- | --- | --- | --- | --- | --- | --- | --- | --- | --- | --- |

|  | 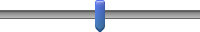 |
| --- | --- |

(Optional) Comments on any of the previous set of 5 questions?

________________________________________________________________

End of Block: Physical Opportunity

Start of Block: Social Opportunity

**What is SOCIAL opportunity?**


Influences from other people, social cues, and cultural norms provide the opportunity to practice healthy eating. Cultural norms can be described as the social setting/environment in which we live, including the social and cultural aspects of life that dictate the way we think about things.


(e.g., Having support from friends and family to practice healthy eating)


**Remember:** HEALTHY EATING supports physical, social, and mental well-being. Healthy eating: includes a variety of vegetables, fruits, whole grains, low-fat dairy or dairy alternative, seafood, legumes, and nuts; is moderate in alcohol; is lower in red and processed meat; and is low in sugar-sweetened foods and drinks and refined grains.

Q29 I have the SOCIAL opportunity that I need for healthy eating.

- Strongly disagree
- Somewhat disagree
- Neither agree nor disagree
- Somewhat agree
- Strongly agree

Q30 In general, my friends are supportive of my healthy eating practices.

- Strongly disagree
- Somewhat disagree
- Neither agree nor disagree
- Somewhat agree
- Strongly agree

Q31 In general, my family is supportive of my healthy eating practices.

- Strongly disagree
- Somewhat disagree
- Neither agree nor disagree
- Somewhat agree
- Strongly agree

| Page Break |  |
| --- | --- |

Q32 The people who I spend time with normally practice healthy eating.

- Strongly disagree
- Somewhat disagree
- Neither agree nor disagree
- Somewhat agree
- Strongly agree

Q33 Healthy eating is common for the groups of people with whom I feel most connected.

- Strongly disagree
- Somewhat disagree
- Neither agree nor disagree
- Somewhat agree
- Strongly agree

Q34 I do NOT feel that I have the social opportunity for healthy eating.

- Strongly disagree
- Somewhat disagree
- Neither agree nor disagree
- Somewhat agree
- Strongly agree

| Page Break |  |
| --- | --- |

Overall, did you find the previous set of 6 questions difficult or easy to read?

|  | Difficult to read | Easy to read |
| --- | --- | --- |

|  | 0 | 1 | 2 | 3 | 4 | 5 | 6 | 7 | 8 | 9 | 10 |
| --- | --- | --- | --- | --- | --- | --- | --- | --- | --- | --- | --- |

|  | 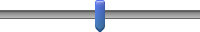 |
| --- | --- |

Overall, how confident are you that you understood what the previous set of 6 questions were asking?

|  | Not at all confident | Very confident |
| --- | --- | --- |

|  | 0 | 1 | 2 | 3 | 4 | 5 | 6 | 7 | 8 | 9 | 10 |
| --- | --- | --- | --- | --- | --- | --- | --- | --- | --- | --- | --- |

|  | 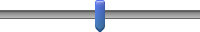 |
| --- | --- |

(Optional) Comments on any of the previous set of 6 questions?

________________________________________________________________

End of Block: Social Opportunity

Start of Block: Demographics

What is your age?

- 65 – 69 years
- 70 – 74 years
- 75 – 79 years
- 80 – 84 years
- 85 years or older
- Prefer not to answer

| Page Break |  |
| --- | --- |

Which describes you? Please select all that apply.

- White
- Black or African American
- Asian
- American Indian or Alaska Native
- Native Hawaiian or Pacific Islander
- Hispanic, Latino, or Spanish origin
- Other (please specify) ________________________________________________

| Page Break |  |
| --- | --- |

Which statement best describes your current employment status?

- Working (paid employee)
- Working (self-employed)
- Not working (temporary layoff from a job)
- Not working (looking for work)
- Not working (retired)
- Not working (disabled)
- Not working (other) ________________________________________________
- Prefer not to answer

| Page Break |  |
| --- | --- |

What is the highest level of school, college or vocational training that you have finished?

- Less than 9th grade
- 9–12th grade, no diploma
- High school graduate (or GED/equivalent)
- Associate's degree or vocational training
- Some college (no degree)
- Bachelor's degree
- Graduate or professional degree

| Page Break |  |
| --- | --- |

What is your gender?

- Male
- Female
- Prefer to self-describe ________________________________________________

End of Block: Demographics

###### Focus Group Guide
